# Self-Nanoemulsion Loaded with a Combination of Isotretinoin, an Anti-Acne Drug, and Quercetin: Preparation, Optimization, and In Vivo Assessment

**DOI:** 10.3390/pharmaceutics13010046

**Published:** 2020-12-30

**Authors:** Khaled M. Hosny, Khalid S. Al Nahyah, Nabil A. Alhakamy

**Affiliations:** 1 Department of Pharmaceutics, Faculty of Pharmacy, King Abdulaziz University, Jeddah 21589, Saudi Arabia; Kabdullahalnahyah@stu.kau.edu.sa (K.S.A.N.); nalhakamy@kau.edu.sa (N.A.A.); 2 Center of Excellence for Drug Research and Pharmaceutical Industries, King Abdulaziz University, Jeddah 21589, Saudi Arabia

**Keywords:** isotretinoin, acne, nanoemulsion, hepatoprotective, Box–Behnken Design

## Abstract

Acne vulgaris is a common skin disease that affects everybody at least once in their lives. The treatment is challenging because the stratum corneum contains rigid corneocytes surrounded by intercellular lamellae that are difficult to bypass. In the present study, we intended to formulate an effective nanoemulsion that could deliver isotretinoin (ITT) with enhanced solubility, permeability, and bioavailability across the skin. ITT can have a serious hepatotoxic effect if given too frequently or erratically. Therefore, to overcome the aforesaid limitation, quercetin (QRS), a hepatoprotective agent, was incorporated into the formulation. Initially, the ITT solubility was determined in various surfactants and cosurfactants to select the essential ingredients to be used in the formulation and to optimize a nanoemulsion that could enhance the solubility and permeability of ITT and its antimicrobial activity against *Staphyloccocus aureus*, which is the main microorganism responsible for acne vulgaris. The mixture design was applied to study the interactions and optimize the independent variables that could match the prerequisites of selected dependent responses. A formulation containing 0.25 g of rosehip oil, 0.45 g of surfactant (Lauroglycol-90), and 0.3 g of cosurfactant (propylene glycol) was chosen as an optimized desirable formulation. The optimized batch was loaded with QRS and evaluated for in vitro and ex vivo permeation. The in vivo hepatotoxicity was assessed through topical administration. Permeability studies confirmed the enhanced permeation percentage of ITT (52.11 ± 2.85%) and QRS (25.44 ± 3.18%) of the optimized formulation, with an enhanced steady-state flux (Jss). The in vivo studies conducted on experimental animals demonstrated superior hepatoprotective activity of the prepared optimized formulation compared with other formulations of drugs and commercially marketed products. We anticipate that this optimized ITT formulation, followed up with good clinical evaluations, can be a breakthrough in the safe treatment of acne vulgaris.

## 1. Introduction

Acne is the common skin disease for which most patients attempt to get a dermatologic cure [1,2]. Recently, dermatological and cosmetic efforts have begun to concentrate on the microbiome of the human skin. *Staphylococcus aureus* adapts to evolving microenvironments of the skin and may become an opportunistic pathogen causing acne or atopic dermatitis [3]. *S. aureus* is a gram-positive, anaerobic organism that usually causes superficial sebaceous unit infections [4]. However, the precise contribution of *S. aureus* to acne is not entirely clear [5]. In chronic inflammatory skin diseases, the microbe can amplify symptoms. The first step in patients is to elucidate their potential role in skin diseases compared with controls. This could lead to new options for more focused antimicrobial therapy [6]. Acne treatment goals include scar reduction and the resolution of lesions. Acne grading depending on the form and severity of the lesions will help guide care [7].

The most commonly used drug for acne is isotretinoin (ITT), which is a synthetic form of vitamin A. It is very effective for mild nodular and extreme papulopustular acne [8]. ITT effectively addresses all aspects of acne pathogenesis; it not only decreases keratinocyte proliferation and thus reduces follicular hyperkeratinization, but also relieves sebaceous gland inflammation and slows sebum development [9]. The pharmacological profile of ITT suggests that it acts primarily by reducing the size of sebaceous glands and the production of sebum, and as a result, it alters the skin surface lipid composition. Bacterial skin microflora are reduced, probably as a result of altered sebaceous factors. ITT 1 to 2 mg/kg/day for 3 to 4 months produces 60% to 95% clearance of inflammatory lesions in patients with severe, recalcitrant nodulocystic acne, and there is evidence of continued healing and prolonged remissions in many patients after treatment withdrawal. In 2016, the American Academy of Dermatology approved ITT for the treatment of moderate acne that either produces physical scarring or is treatment resistant [10,11]. The low oral bioavailability of ITT in animals can result from the biodegradation of the drug in the gastrointestinal (GI) lumen and/or drug metabolism during absorption (in the GI mucosa) and the first passage through the liver. ITT can have a significant effect on the population of resident microbes by eliminating their supply of critical nutrients and stabilizing immune hypersensitivity [12]. Over the decades, no other drugs or treatments have been as significantly effective as ITT. Nonetheless, despite these fascinating features, the drug’s extremely low solubility and permissibility can limit its incorporation into a suitable vehicle and lead to poor patient compliance. ITT can cause a dose-related elevation of blood triglyceride and cholesterol levels; it increases the level of triglyceride in 50% of patients and cholesterol in 30%. Ahmadvand et al. showed that before and after ITT therapy, both triglyceride and cholesterol levels had significant statistical differences [13]. ITT can interact with certain essential groups at the active sites of important proteins or enzymes in the lipid metabolism, such as hydroxymethylglutaryl (HMG), a key regulatory enzyme that plays an important role in the metabolism of cholesterol. ITT can also cause an elevation in the liver transaminases in a dose-dependent manner during treatment [13].

Consequently, it is prudent to enhance the solubility of ITT and minimize its adverse effects by using an appropriate carrier.

Several plant-derived natural products, characterized by a huge structural diversity, including phenolic compounds, are cited as antimicrobial agents and resistance-modifying agents [14]. One such compound is quercetin, which comprises a variety of phenolic hydroxyl groupings with significant anti-inflammatory properties and antioxidant effects [15]. Quercetin was reported to have strong, long-lasting anti-inflammatory effects [16]. Its anti-inflammatory potential can be expressed on different cell types in animal and human models (Chirumbolo, 2010). The QRS mainly remained in the layers of the human stratum corneum. The properties of quercetin have potential benefits for overall health and disease resistance, including anticarcinogenic [17], anti-inflammatory, antiviral, antioxidant, and psychostimulant benefits. The drug can also inhibit lipid peroxidation, platelet aggregation, and capillary permeability and can stimulate mitochondrial biogenesis [18,19,20]. Quercetin’s antioxidant features can assist in the care and appearance of the skin [21]. The key downside of quercetin applications via nanodelivery techniques is stability and effectiveness of penetration. We conclude that quercetin can be used to make different cosmeceutical nanosized products with improved stability and sustained beautifying effects and no harmful side effects [22].

The self-nanoemulsifying drug delivery system (SNEDDS) is a nanocarrier that forms spontaneously from the mixture of an oil, surfactant, cosurfactant, and active drug compound in a homogenous, fine, oil-in-water, nanoemulsion liquid form [23].

In recent years, much attention has been paid to SNEDDS. The systems have had reasonable success in improving the bioavailability of poorly soluble drugs, especially topical drugs. Nanoemulsions have also been used for the decontamination of radionuclei and photodynamic therapies. Consequently, nanoemulsions have many applications in the field of dermatology [24]. SNEDDS have a broad interfacial surface area because of their small globule size, and this enhances drug absorption and bioavailability by improving drug release and membrane permeation and reducing presystem metabolism and efflux pumping [25]. Many therapeutic agents, such as antioxidants, nonsteroidal anti-inflammatory drugs, and lipids were successfully formulated using nanoemulsions as novel carriers for topical application. Consequently, nanoemulsions have many applications in the field of dermatology [26].

Numerous scientific studies were done over the years on the application of essential oils in nanoemulsions. An essential oil can be a suitable natural alternative to a synthetic skin penetration enhancer due to its low cost and its ability to improve penetration [27]. Rosehip oil extracted from *Melaleuca alternifolia* has a unique application in the treatment of acne. Owing to its improved bacteriostatic activity and ability to minimize lesion formation, rosehip oil has been used to treat seborrheic dermatitis and acne vulgaris. There are several drawbacks related to its topical application, such as the potential production of irritant or allergic contact dermatitis, decreased penetration in the affected area, and potential alteration of cutaneous flora [28]. It was allegedly used in Persian medicine to relieve inflammation, cure infectious diseases, and heal wounds [29]. In the treatment of acne, rose oil was used; it is a multifunctional component that may be integrated into cosmetic products [30,31].

In contrast to several conventional methods, the topical route offers an alternative form of drug delivery and attracts innovative research, particularly in the treatment of several skin diseases. The potential nanotechnology strategy circumvented the physicochemical and pharmacokinetic constraints of QRS and ITT. Nonetheless, very few studies have discussed the crucial findings of the in vivo results of this advanced method. Therefore, this study used a mixture design to create an optimized formulation of quercetin and ITT to improve their bioavailability and antimicrobial activity and to reduce ITT toxicity.

## 2. Materials and Methods

### 2.1. Materials

ITT was purchased from Wuhan Senwayer Century Chemical Co., Ltd. (Wuhan, China). Rosehip oil was obtained from Hayward, CA, USA. Quercetin was procured from Water Import and Export Co., Ltd. (Exiamen, China). Lauroglycol-90 was generously gifted by Gattefosse (Lyon, France). Propylene glycol, Tween, Span, and Cremophor were purchased from Sigma (St. Louis, MO, USA). All other reagents and chemicals used were of analytical grades.

### 2.2. Methods

#### 2.2.1. Determination of ITT Solubility

The solubility of ITT in rosehip oil and different surfactants and cosurfactants was determined. An excess amount of ITT was dissolved in approximately 3 mL of each vehicle individually. The mixture was then shaken on a water bath (Model-1031, GFL Corporation, Lüneburg, Germany) for 72 h, and the temperature was maintained at 37 ± 0.5 °C. Once equilibrium was attained, the resulting mixture was centrifuged (3K30 Centrifuge, Sigma Aldrich, Gillingham, Dorset, UK) at 8 °C for 30 min at 15,000 rpm. The formed supernatant was diluted with a suitable quantity of methanol and analyzed quantitatively for the ITT concentration using a UV-Visible spectrophotometer at 362 nm. The experiment was repeated three times, and the values were noted as the mean ± the standard deviation.

#### 2.2.2. Pre-Formulation Studies

Several formulations were designed with selected proportions of rosehip oil, Lauroglycol-90 surfactant, and propylene glycol cosurfactant and measured for particle size using the Zetatrac particle size analyzer (Microtrac, Inc., Montgomeryville, PA, USA) in accordance with the study design. Initially, 10 formulations were prepared (Table 1) using different concentrations of ITT (20 to 60 mg) and different proportions of rosehip oil, Lauroglycol-90, and propylene glycol. The amount of QRS in each formula was equal to ITT. QRS at lower doses can be very useful in maintaining human health; at higher doses, QRS has proapoptotic actions on healthy cells but can kill tumor cells.

#### 2.2.3. Mixture Design for the Development of ITT-QRS-Loaded SNEDDS

Statistical optimization of the response surface methodology (RSM) has proven to be a proficient tool in optimizing the various parameters in a multifarious process to obtain the desired responses; thus, it has been applied to formulate ITT-QRS SNEDDS. Among several models, the I-optimal point-exchange mixture design was selected to study the interaction of independent variables. Three factors were selected, namely, the percentage of rosehip oil (X1), surfactant (Lauroglycol-90) (X2), and cosurfactant (propylene glycol) (X3) (Table 2). Data from the previous pre-formulation studies were used to choose the levels of independent variables. All these components were used selectively in several ratios, with a total concentration equal to 100%. A total of 16 runs were projected, and the variables are summarized in Table 3. All these variables were selected with regard for the fact that the developed formulation was specially meant for topical application. The globule size (Y1), steady-state inflow (Y2), and inhibition zone (Y3) play vital roles in the delivery of active ingredients to the site of action. The correlations between the selected variables and responses were further analyzed by applying the regression equation using Statgraphics Centurion XV software version 15.2.05 (StatPoint Technologies, Warrenton, VA, USA). The SNEDDS formulation was optimized for the minimum globule size, highest Jss, and maximum inhibition zone.

#### 2.2.4. Preparation of ITT-QRS Nanoemulsion

**Figure 1:**
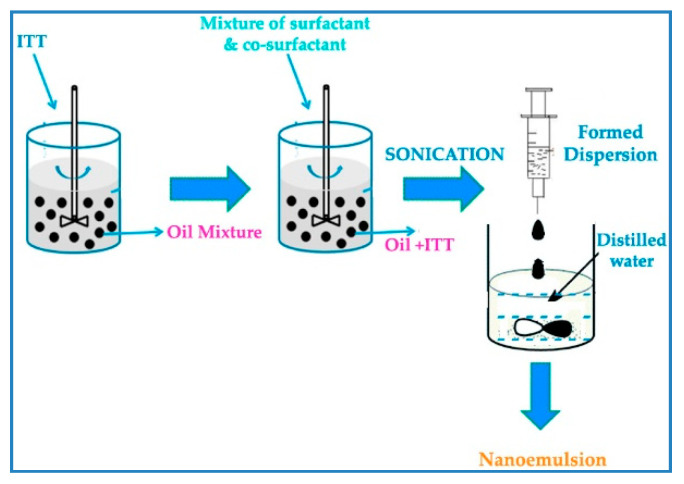
Preparation of ITT nanoemulsion.

Various formulations of ITT-QRS nanoemulsions were prepared, as portrayed in the design mixture (Figure 1). First, ITT and QRS were added to the rosehip oil; subsequently, a mixture of Lauroglycol-90 and propylene glycol was added. The formed mixture was further sonicated using Ultrasonic Processors (VCX 750, Sonics & Materials, Inc., Newtown, CT, USA) to aid in the dissolution of ITT within the formed mixture. The formed mixture was homogenized for 3 min using a 1.3-mm stainless steel probe. At this stage, the formulation could be further loaded with quercetin as required. Finally, the formed dispersion (self-nanoemulsifying system) was added to distilled water drop by drop, and the final nanoemulsions were formed spontaneously during mixing at room temperature (Figure 1).

#### 2.2.5. Characterization of ITT-QRS Nanoemulsion

Prepared ITT-QRS SNEDDS were characterized by various parameters to study the effect of selected variables.

##### Measurement of Globule Size

For each formulation, 100 μL of the formulation was diluted with 900 μL of distilled water to form an aliquot of 1 mL, and the globule size of the prepared sample was determined at 25 °C using a dynamic light-scattering technique (Zetatrac, Microtrac, Montgomeryville, PA, USA) [30].

##### Ex Vivo Permeation Studies to Evaluate the Jss of ITT from Each Formula

The ex vivo permeation test was done with a simple, low-head dissolution testing instrument (PT-DT70, Pharma Test Apparatebau AG, Hainburg, Germany). The receptor compartment was filled with 450 mL of phosphate buffer with continuous stirring at 50 rpm. The temperature of the buffer solution was maintained at 37 ± 0.5 °C. The required area of skin (2 cm × 2 cm) was collected from a Wistar rat’s abdominal segment (hairless). Animals were collected from the Animal House at the Beni-Suef clinical laboratory center after obtaining approval from the ethical committee (Approval No. 22-09-20). Obtained skin was soaked in a phosphate buffer at a pH of 7.2 for 1 day before the study began. Afterward, the skin segment was placed between the donor and receptor compartments so that the dermal side would be in close contact with the receptor side. At specific intervals, aliquots of 3-mL samples were withdrawn, and the value of the Jss for each formula was analyzed using a UV-Visible spectrophotometer (Jenway 6705, Cole-Parmer, Stone, Staffordshire, UK). The QRS and ITT were quantitatively measured at 269 nm and 299 nm, respectively.

##### Inhibition Zone against *Staphylococcus aureus*

An antibacterial activity test assessing the inhibition zones produced by the test formulations was performed by plating 20 mL of agar (cooled) plus an *S. aureus* inoculum into a petri dish. When set, the agar was punched with a sterile cork-borer 12 mm in diameter, and 100 μL of each formulation was added to the bores. After incubation at 37 °C for 18 h, the inhibition zones were measured with a ruler (*n* = 3). Sterile water was used as a negative control.

#### 2.2.6. Optimization of ISN Formulations

The obtained results from the experimental runs were further analyzed using the ANOVA (analysis of variance) with a multiple-response optimization. The optimized formulation was selected based on the constraints of the selected responses.

#### 2.2.7. Preparation and Evaluation of the Optimum Formula According to the Mixture Design

After the optimization of the experimental runs, an optimized formulation was prepared according to the concentrations proposed by the software and evaluated for globule size, ex vivo permeation, and inhibition zone against *S. aureus*. In addition, the following tests were done on the optimized formula.

#### 2.2.8. In Vitro Release Studies

In vitro drug release studies were performed to calculate the amount of ITT and QRS released in the optimized formulation. A low-head dissolution testing instrument, PT-DT70, with a dialysis cellulose membrane diffusion area of 4 cm^2^ (Cutoff 14,000 Da pore size; Sigma-Aldrich, St Louis, MO, USA) was utilized. First, the dialysis membrane was soaked in a phosphate buffer (pH 7.4) for 15 min at 25 ± 0.5 °C. The receptor compartment was filled with the phosphate buffer, and the paddle was placed in the medium with continuous rotations (75 rpm). The temperature was maintained at 37 ± 0.5 °C throughout the experiment. Samples were withdrawn at various time intervals (1, 2, 3, 4, 6, 8, and 12 h), and the ITT and QRS concentrations were analyzed at 269 and 299 nm, respectively.

#### 2.2.9. Animal Studies

**Figure 2:**
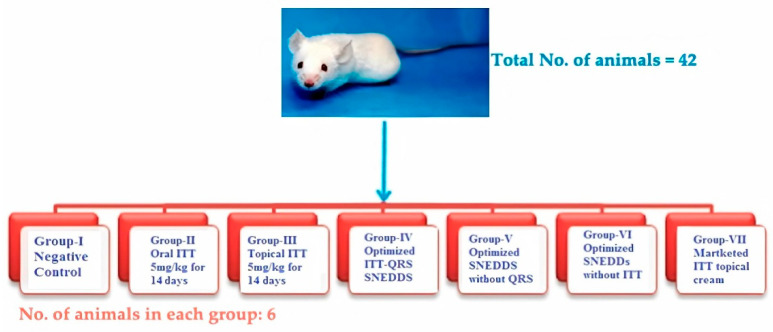
In Vivo animal study groups and sample information.

Forty-two male adult mice weighing 20.5 ± 1.5 g were selected and adapted for 7 days before the study. The animals were divided into seven groups consisting of six animals in each group. All groups and the tested formulations are shown in Figure 2. At the end of the study period, the hepatic function parameters were estimated by measuring the AST, ALT, malondialdehyde (MDA), and glutathione peroxidase (GSH-Px).

#### 2.2.10. Statistical Analysis

The in vivo results obtained were expressed as the mean ± the standard deviation and analyzed using the Statistical Package for the Social Sciences version 26 (IBM, SPSS, Ithaca, NY, USA). A one-way ANOVA was followed by a post-hoc Tukey test to determine the significant differences between the quantitative variables. A *p*-value of less than 0.05 was noted as statistically significant.

## 3. Results and Discussion

### 3.1. ITT Solubility Studies

**Figure 3:**
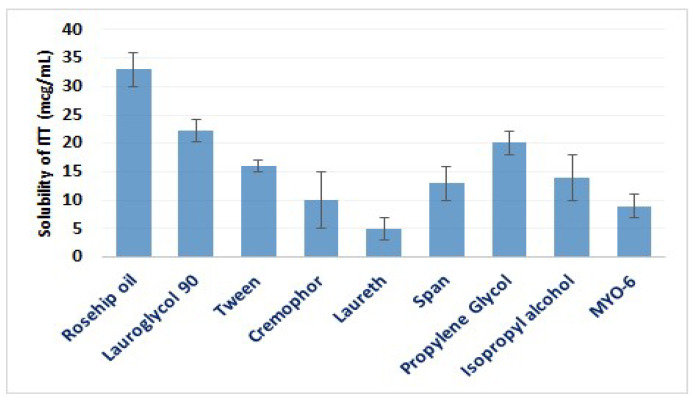
The solubility of ITT in assorted vehicles. Data are presented as mean ± SD (*n* = 3).

The solubility of ITT in assorted vehicles is depicted in Figure 3. The highest level of ITT solubility was 22.33 ± 4.22 µg/mL, and it was found with Lauroglycol-90 (a nonionic emulsifier); comparable results were observed with other drugs with low solubility, as specified by Patel et al. [32]. Hence, Lauroglycol-90 was the best carrier for increasing the solubility of hydrophobic drugs. Additionally, Lauroglycol-90–based microemulsions were formulated for topical application [33]. Surfactants generally act as penetration enhancers because they can inhibit the P-glycoprotein efflux transporters, increasing the permeation of several drugs. Lauroglycol-90 can also cause fluidization of cell lipoidal membranes to open the tight junctions, and this enhances permeability. Hence, the dual properties of Lauroglycol-90 as a solubilizer for hydrophobic drugs such as ITT and a penetration enhancer make it the most productive element in improving the permeability of ITT.

The enhanced solubility of ITT has been recorded with propylene glycol (20.1 ± 3.11 µg/mL). Propylene glycol is known for its great miscibility in polar and nonpolar solvents, as well as its ability to increase absorption and the degree of permeability [34]. Thus, it serves as a carrier (cosurfactant) in formulating nanoemulsions. Propylene glycol can penetrate and interact with the stratum corneum to modify the permeability of several therapeutic agents. Several studies have confirmed the enhanced solubility and permeability of propylene glycol as a vehicle [35]. Rosehip oil also solubilizes ITT because it is a hydrophobic “drug” to a reasonable extent. Based on these results, the surfactant Lauroglycol-90, cosurfactant propylene glycol, and essential oil rosehip oil were selected to formulate the SNEDDS.

### 3.2. Pre-Formulation Studies

The particle sizes of all the prepared formulations were evaluated. First, the concentration of ITT to be used for further formulations was optimized. Formulation 1, having higher concentrations of ITT (20, 40, and 60 mg), resulted in a particle size of 114.11 ± 13.28 nm, 186.22 ± 13.62 nm, and 350.44 ± 21.32 nm (Table 4). Therefore, a concentration of 20 mg of ITT was selected in the research. The identical phenomenon was observed with formulations 2 and 3. Among the selected doses of ITT, a 20-mg daily dose seems to be an effective and safe treatment option for patients with moderate to severe acne, with a lower incidence of side effects. To the best of our knowledge, there is no reported use in the literature of a fixed 20-mg daily ITT dose until a cumulative dose of 0.5–1 mg/kg has been reached. This was supported by the work done by Abbas Rasi et al. As a result, a concentration of 20 mg of ITT was used consistently to prepare the remaining formulations. And in each tested formula amount of QRS was equal to that of ITT [1:1 ratio of ITT:QRS]. (Table 4) [36].

### 3.3. Optimization of ITT-Loaded SNEDDS

The mixture design was used to analyze the effect of selected independent variables on the minimum globule size (Y1), maximum Jss (Y2), and larger inhibition zone (Y3). The design projected 16 runs, and the observed responses are given in Table 5. The globule size in all the trial batches was found to be 130 to 390 nm. The Jss and inhibition zone were estimated to be 95 to 270 µg/cm^2^/h and 3 to 27 mm, respectively (Table 5). All obtained results were evaluated for individual responses. The effect of selected variables was further analyzed statistically using the ANOVA.

Based on the fit summary of the responses (adjusted and predicted R2 values) and the sequential sum of squares (Type I), different models were selected (Table 6). No models were misidentified with the highest-order polynomial equations [37]. The accuracy of the model was further evaluated using the normal probability of studentized residuals. Externally studentized results were scattered around the straight line with a slight deviation. The ANOVA was further used to quantitatively analyze the relationship between the selected independent variables and the responses.

Response I: The selected model was statistically significant, F = 32, *p* < 0.01%. Polynomial equations were generated using multiple regression analysis. Both the *p*-values and polynomial equations were used to estimate the true effect of the variables. *p*-values that were less than 0.0001 were considered statistically significant. Values greater than 0.1000 indicated that the model terms were not significant. If there were many insignificant model terms (not counting those required to support the hierarchy), model reduction could have been required. The lack-of-fit F-value (0.52) was nonsignificant, with only a 13.5% chance that a lack-of-fit F-value this large could occur due to noise.

Adequate precision measured the signal-to-noise ratio. In general, a ratio greater than 4 is desirable. The obtained ratio (194.91) indicated an adequate signal to navigate the design space [38]. ANOVA results revealed the significant statistical relationship between the components and responses at a 95% confidence level.

Based on the ANOVA results, Response I was affected significantly by the synergistic effect of a linear mixture (*p* < 0.0001) between rosehip oil and other factors, but the predominant effect was for rosehip oil [39,40] with the highest magnitude (Table 7). The polynomial equation can be further applied to predict the response from any given concentration of independent variables. The equation was generated at the point where lower concentrations of independent factors could contribute to the formation of globules with a minimum size.

Final Equations with Coded Factors

Globule size (Y1) = +157.19 X1 − 1011.05 X2 + 1161.66 X3 + 3067.47 X1X2 − 1077.94 X1X3 + 222.78 X2X3 − 2252.11 X1X2X3

**Figure 4:**
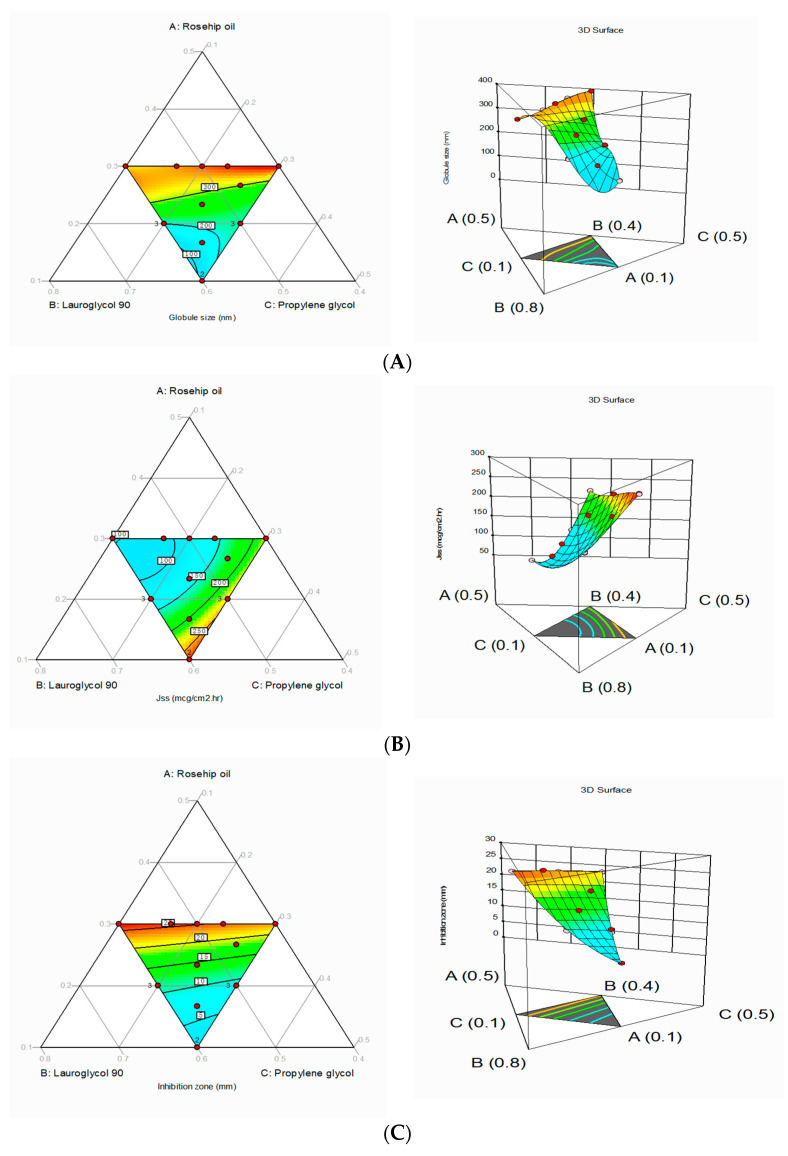
Contour and three-dimensional response surface graphs for (**A**) globule size, (**B**) Jss, and (**C**) inhibition zone.

Several studies have reported that increased oil levels can increase the globule size. This can be due to the coalescence of oil droplets and the added concentrations of surfactants or cosurfactants. Conversely, increasing the proportion of Lauroglycol-90 can reduce the globule size. RSM has been applied to analyze the effects of these selected factors. Contour plots and three-dimensional graphs for Response I are shown in Figure 4. The mixture’s three components are depicted in the corners of the triangle, and the center portion illustrates the mixture.

Response II: The model F-value (11.08, *p* < 0.01) and lack-of-fit F-value (4.36) for Response II confirmed that the selected model was significant, and the lack of fit was not significant, relative to the pure error. Adequate precision (84.54) indicated an adequate signal to navigate the design space. Based on the ANOVA results (Table 7) for the cubic model, X1, X3, X2X3, X1X2X3, and X2X3 (X2 to X3) factors affected Response II antagonistically at a *p*-value of less than 0.0001, and all remaining factors showed a synergistic effect (Table 4). The generated polynomial equation shows that all these selected variables significantly affected the Jss. The mixture design contour plots and three-dimensional plots were similar to those for Response I. As discussed in the previous sections, higher concentrations of surfactants and rosehip oil account for an elevated and decreased Jss, respectively.

Final Equations with Coded Factors

Jss (Y2) = +497.49 X1 + 408.09 X2 + 915.05 X3 − 1382.62 X1X2 − 1944.53 X1X3 − 1569.70 X2X3

Response III: The adequate precision for the linear model of Response III was 36.366, which is greater than 4, suggesting that the model could navigate the design space. The ANOVA results confirmed the significant effect of the linear mixture (*p* < 0.0001). The three selected components are depicted in the corners of the triangle, and the three-dimensional contour plots were drawn to study the interaction effects. The fitted linear special regression equation was generated as shown below.

Final Equations with Coded Factors

Inhibition zone = 78.95 X1 + 8.39 X2 − 4.30 X3 − 66.65 X1X2 − 57.07 X1X3 + 3.12 X2X3

The above results showed a higher significant effect of the rosehip oil concentration compared with the other variables. This was due to the antimicrobial properties of the rosehip oil [41,42].

**Figure 5:**
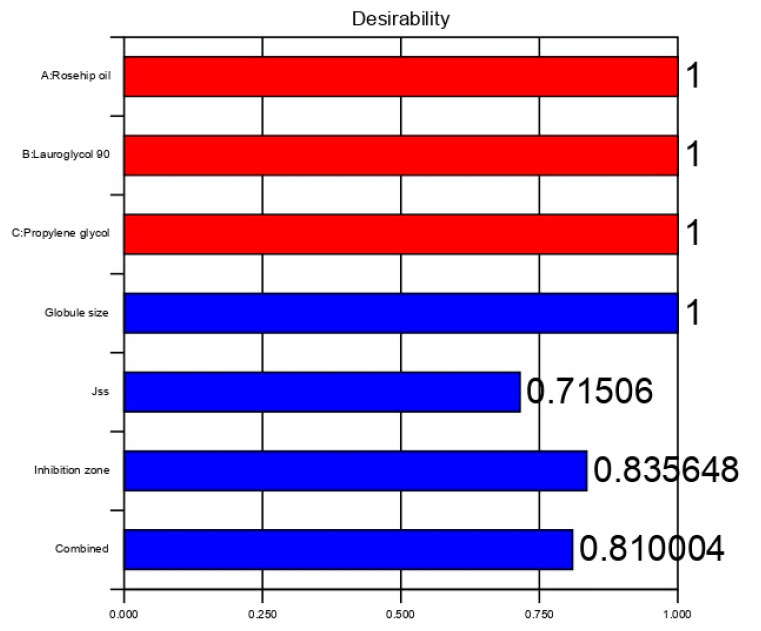
Bar chart showing the desirability of the selected design.

The process was optimized by setting the goals for every response and simultaneously applying the global desirability function (D). Based on these criteria, the desirability plot was generated with a D value of 1 (Figure 5). In conclusion, a formulation with 0.25 g of the oil mixture, 0.45 g of the surfactant (Lauroglycol-90), and 0.3 g of the cosurfactant (propylene glycol) fulfilled the optimum formulation requirements. Using these variables can result in a formulation with a globule size of 249 nm, Jss of 222.05 µg/cm^2^/h, and inhibition zone of 15 mm.

### 3.4. Validation of Experimental Design

In the end, the experimental design was further validated to confirm its accuracy. The optimized formulation was prepared with the given conditions and evaluated for the globule size, Jss, and inhibition zone. The relative error was calculated by comparing the predicted and practical values, as shown in Table 8. The relative error was within the acceptable range (±5%), thus confirming the design’s preciseness. Generally, emulsions are thermodynamically stable isotropic systems in which two immiscible liquids are mixed to form a single phase utilizing an emulsifying agent, that is, a surfactant and cosurfactant. The droplet size of the nanoemulsion is typically in the range 20 to 300 nm for an optimized penetration with topical application. The results of the optimized formula showed a globule size of 245 nm which was in agreement with previously published data which indicated that, the globular size for any transdermal and topically applied vesicular drug delivery system is preferred to be less than 300 nm in order to penetrate easily through skin layers [42,43].

### 3.5. In Vitro Permeation/Release Study

**Figure 6:**
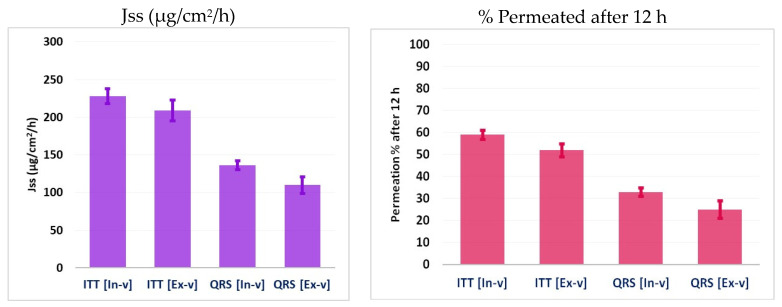
In Vitro [In-v] and ex vivo [Ex-v] permeation and Jss results of the optimized formulation loaded with ITT-QRS SNEDDS. Data presented as the mean ± standard deviation. (*n* = 3). The level of significance was considered at *p* < 0.05.

The percentage of permeability through the cellulose membrane for the QRS and ITT was found to be 33.35 ± 2.17% and 59.43 ± 4.23% after 12 h (Figure 6). The Jss values further supported this finding. ITT had more Jss values, resulting in a higher level of permeation.

### 3.6. Ex Vivo Permeation Study

Ex vivo studies were conducted using a simple, low-head dissolution apparatus to establish the effect of the ITT-QRS formulations. The permeation was studied using rat full-thickness skin, and the results are summarized in Figure 6. An increased percentage of ITT permeation (52.11 ± 2.85%) was observed for the optimized nanoemulsion formulation. This can be credited to the synergistic action of Lauroglycol-90 and propylene glycol in fluidizing the cell membranes and inhibiting the P-glycoprotein efflux. Another factor is the high level of permeation of nanoemulsion drug delivery systems. Nanoemulsions have proven to have relevance in clinical applications owing to their increased drug loading, enhanced drug solubility and bioavailability, reduced patient variability, controlled drug release, and protection from enzymatic degradation. A nanoemulsion can act like a drug reservoir in which the loaded drug is released into the outer phase and then into the skin.

### 3.7. In Vivo Studies

Despite several notable advancements, hepatic safety and hepatic cell regeneration products are inadequate for treating several liver disorders. Numerous herbal extract formulations were also used in this approach [44,45]. QRS is a natural flavonoid with exceptional antioxidant properties [46,47,48,49]. A significant alteration in hepato-specific liquid peroxidation and oxidative stress was observed with ITT. A similar result was observed in a few previous studies [50,51]. Table 9 shows the elevation of ALT and AST levels observed with formulations containing ITT alone.

Consistent with these results, a significant rise in the MDA levels and lipid peroxidation and a reduction in the antioxidant enzymes were supported by a significant decrease in the GSH-Px level.

Pretreatment with a formulation containing ITT plus quercetin for 14 days significantly increased the antioxidant characteristics and reduced the amount of lipid peroxidation, and this reduced the overall oxidative stress. All changes obtained with the QRS formulation (Group 4) were more beneficial in ameliorating hepatotoxicity compared with the formulation without QRS (Group 5). Additionally, Group 4 differed significantly from the marketed product (Group 7). No significant differences (*p* > 0.05) emerged in Group 5, which was treated with the formula that lacked QRS and the commercially available ITT formulation, and Group 3, which was treated with topical ITT in an aqueous dispersion, for all tests.

## 4. Conclusions

In the present study, a topical nanoemulsion of ITT was successfully optimized in conjunction with QRS to enhance the permeability and to study the relevance of hepatoprotective agents from the formulation. ITT exhibited maximum solubility in the Lauroglycol-90 surfactant and propylene glycol cosurfactant, and this further optimized using the quality by design desirability approach (mixture design). A lower concentration of ITT favored small vesicle formation. The optimum formulation consisted of 0.25 g of rosehip oil, 0.45 g of a surfactant (Lauroglycol-90), and 0.3 g of a cosurfactant (propylene glycol). The range of relative error was acceptable (<5%), confirming the preciseness of the design. In vitro and ex vivo permeation studies confirmed the enhanced percentage of permeation of ITT and QRS, and in vivo studies further supported these results. The QRS had commensurate hepatoprotective activity in the prepared formulation and was found to be safe in comparison with formulations containing individual drugs and commercially marketed products. However, further clinical investigations are required to explore the commercial viability of the product.

## Figures and Tables

**Table 1:** Preliminary formulation prepared to study the emulsification of isotretinoin (ITT) and quercetin (QRS).

Formula	Formulation
1	Rosehip oil (0.2 g) + Lauroglycol-90 (0.6 g) + Propylene glycol (0.2 g) + 20 mg ITT+ 20 mg QRS
2	Rosehip oil (0.2 g) + Lauroglycol-90 (0.6 g) + Propylene glycol (0.2 g) + 40 mg ITT+ 40 mg QRS
3	Rosehip oil (0.2 g) + Lauroglycol-90 (0.6 g) + Propylene glycol (0.2 g) + 60 mg ITT+ 60 mg QRS
4	Rosehip oil (0.3 g) + Lauroglycol-90 (0.6 g) + Propylene glycol (0.1 g) + 20 mg ITT+ 20 mg QRS
5	Rosehip oil (0.3 g) + Lauroglycol-90 (0.4 g) + Propylene glycol (0.3 g) + 20 mg ITT+ 20 mg QRS
6	Rosehip oil (0.1 g) + Lauroglycol-90 (0.7 g) + Propylene glycol (0.2 g) + 20 mg ITT+ 20 mg QRS
7	Rosehip oil (0.1 g) + Lauroglycol-90 (0.5 g) + Propylene glycol (0.4 g) + 20 mg ITT+ 20 mg QRS
8	Rosehip oil (0.2 g) + Lauroglycol-90 (0.6 g) + Propylene glycol (0.2 g) + 20 mg ITT+ 20 mg QRS
9	Rosehip oil (0.3 g) + Lauroglycol-90 (0.6 g) + Propylene glycol (0.1 g) + 20 mg ITT+ 20 mg QRS
10	Rosehip oil (0.1 g) + Lauroglycol-90 (0.5 g) + Propylene glycol (0.4 g) + 20 mg ITT+ 20 mg QRS

**Table 2:** Experimental mixture design (component levels and selected responses).

Component	Level		Response	Constraints
	Low	High		
Rosehip oil (%), (X1)	10	30	Mean globule size (Y1)	Minimum
Lauroglycol-90 (%), (X2)	40	60	Steady-state inflow (Jss) (Y2)	Maximum
Propylene glycol (%), (X3)	10	30	Inhibition zone (Y3)	Maximum

**Table 3:** Mixture design for the development of ITT-QRS-loaded self-nanoemulsifying drug delivery system (SNEDDS).

Run	Independent Variables		
	(X1): Oil (Rosehip Oil)	(X2): Surfactant (Lauroglycol-90)	(X3): Cosurfactant (Propylene Glycol)
1	0.3	0.5	0.2
2	0.2	0.5	0.3
3	0.3	0.533333	0.166667
4	0.2	0.6	0.2
5	0.2	0.6	0.2
6	0.233333	0.533333	0.233333
7	0.166667	0.566667	0.266667
8	0.2	0.5	0.3
9	0.3	0.6	0.1
10	0.1	0.6	0.3
11	0.2	0.5	0.3
12	0.2	0.6	0.2
13	0.266667	0.466667	0.266667
14	0.1	0.6	0.3
15	0.3	0.466667	0.233333
16	0.3	0.4	0.3

**Table 4:** Particle size and ITT content of different formulations prepared during pre-formulation step. Data are presented as mean ± SD (*n* = 3).

Formula	Amount of ITT (mg)	Particle Size ± SD (nm)
1	20	114.11 ± 13.28
2	40	286.22 ± 15.62
3	60	350.4 ± 21.32
4	20	311.21 ± 12.33
5	20	297.22 ± 11.11
6	20	91.12 ± 6.75
7	20	81.33 ± 2.76
8	20	130.21 ± 12.3
9	20	350.55 ± 16.1
10	20	100.34 ± 7.80

**Table 5:** Projected trial formulations and their observed responses according to the mixture design.

Run	Globule Size * (nm)	Jss * (µg/cm^2^/h)	Inhibition Zone * (mm)
1	370 ± 13	115 ± 9	25 ± 4
2	220 ± 11	240 ± 13	9 ± 2
3	355 ± 14	95 ± 7	26 ± 6
4	200 ± 10	130 ± 9	11 ± 2
5	199 ± 10	128 ± 6	12 ± 4
6	265 ± 13	135 ± 11	15 ± 2
7	167 ± 14	205 ± 14	6 ± 2
8	218 ± 12	238 ± 12	8 ± 3
9	340 ± 16	107 ± 10	27 ± 5
10	130 ± 9	270 ± 17	3 ± 1
11	217 ± 13	235 ± 23	9 ± 4
12	203 ± 12	127 ± 11	11 ± 2
13	300 ± 14	177 ± 16	19 ± 4
14	132 ± 17	268 ± 12	3 ± 1
15	381 ± 12	140 ± 15	24 ± 5
16	390 ± 18	220 ± 18	23 ± 2

* Average ± SD (*n* = 3).

**Table 6:** Fit Summary for responses.

Source	Globule Size		Jss		Inhibition Zone	
	Adjusted R^2^	Predicted R^2^	Adjusted R^2^	Predicted R^2^	Adjusted R^2^	Predicted R^2^
Linear	0.9455	0.9217	0.8463	0.7051	0.9420	0.9160
Quadratic	0.9933	0.9896	0.9943	0.9887	0.9948	0.9937
Special Cubic	0.9926	0.9783	0.9950	0.9695	0.9950	0.9881
Cubic	0.9996		0.9989		0.9968	

**Table 7:** ANOVA results in three responses.

Term	Responses					
	Globule Size		Jss		Inhibition Zone	
	F-Value	*p*-Value	F-Value	*p*-Value	F-Value	*p*-Value
Model	4645.30	<0.0001	928.75	<0.0001	574.28	<0.0001
Linear Mixture	19,919.44	<0.0001	3223.03	<0.0001	1368.24	<0.0001
AB	74.69	0.0001	18.73	0.0034	74.79	<0.0001
AC	30.07	0.0015	42.96	0.0003	55.22	<0.0001
BC	1.30	0.2975	29.73	0.0010	0.1197	0.7365
ABC	30.87	0.0014	0.0041	0.9510	---	---
AB (A to B)	154.29	<0.0001	6.63	0.0367	---	---
AC (A to C)	165.73	<0.0001	20.28	0.0028	---	---
BC (B to C)	13.88	0.0098	928.75	<0.0001	---	---

**Table 8:** Predicted versus experimental values for selected responses.

Number.	Parameter	Predicted Values	Experimental Values	Relative Error (%)
1.	Globule size (nm)	249	245	1.6
2.	Jss (µg/cm^2^/h)	222.05	228	–2.4
3.	Inhibition zone (mm)	15	14.5	0.4

**Table 9:** The effect of various formulation ingredients on hepatoprotective activity in comparison with the marketed formulation.

Groups	AST	ALT	MDA	GSH
	Mean ± SD	Mean ± SD	Mean ± SD	Mean ± SD
G1 [Control normal saline]	19.8 ± 1.89	21.85 ± 1.47	0.682 ± 0.029	636.47 ± 16.56
G2 [Distilled water + ITT (Oral)]	38.5 ± 3.26	46.57 ± 4.54	1.278 ± 0.030	383.71 ± 13.8
G3 [Distilled water + ITT (Topical)]	31.5 ± 3.50	38.225 ± 3.76	0.984 ± 0.021	445.09 ± 17.48
G4 [Optimum ITT-QRS SNEDDS]	20.75 ± 2.61	22.035 ± 2.11	0.674 ± 0.033	660.30 ± 22.08
G5 [Optimum ITT SNEDDS without QRS]	29.17 ± 2.62	36.895 ± 2.76	1024 ± 0.021	413.63 ± 25.76
G6 [Optimum ITT SNEDDS without ITT]	19.16 ± 2.09	22.735 ± 1.58	0.696 ± 0.019	697.26 ± 22.08
G7 [Marketed ITT cream]	34.41 ± 3.46	38.26 ± 2.07	0.956 ± 0.025	495.89 ± 19.32

## Data Availability

Not applicable.
